# Multi-locus genome-wide association study of chickpea reference set identifies genetic determinants of *Pratylenchus thornei* resistance

**DOI:** 10.3389/fpls.2023.1139574

**Published:** 2023-03-24

**Authors:** Sonal Channale, John P. Thompson, Rajeev K. Varshney, Mahendar Thudi, Rebecca S. Zwart

**Affiliations:** ^1^ Centre for Crop Health, Institute for Life Sciences and the Environment, University of Southern Queensland, Toowoomba, QLD, Australia; ^2^ Centre for Crop & Food Innovation, Murdoch University, Perth, WA, Australia; ^3^ Department of Agricultural Biotechnology and Molecular Biology, Dr. Rajendra Prasad Central Agricultural University, Pusa, India; ^4^ School of Agriculture and Environmental Science, Faculty of Health, Engineering and Science, University of Southern Queensland, Toowoomba, QLD, Australia

**Keywords:** chickpea, *Cicer arietinum*, root-lesion nematode, *Pratylenchus thornei*, GWAS, candidate resistance genes

## Abstract

*Pratylenchus thornei* is an economically important species of root-lesion nematode adversely affecting chickpea (*Cicer arietinum*) yields globally. Integration of resistant crops in farming systems is recognised as the most effective and sustainable management strategy for plant-parasitic nematodes. However, breeding for *P. thornei* resistance in chickpea is limited by the lack of genetic diversity. We deployed a genome-wide association approach to identify genomic regions and candidate genes associated with *P. thornei* resistance in 285 genetically diverse chickpea accessions. Chickpea accessions were phenotyped for *P. thornei* resistance in replicated glasshouse experiments performed for two years (2018 and 2020). Whole genome sequencing data comprising 492,849 SNPs were used to implement six multi-locus GWAS models. Fourteen chickpea genotypes were found to be resistant to *P. thornei*. Of the six multi-locus GWAS methods deployed, FASTmrMLM was found to be the best performing model. In all, 24 significant quantitative trait nucleotides (QTNs) were identified, of which 13 QTNs were associated with lower nematode population density and 11 QTNs with higher nematode population density. These QTNs were distributed across all of the chickpea chromosomes, except chromosome 8. We identified, receptor-linked kinases (RLKs) on chromosomes 1, 4 and 6, GDSL-like Lipase/Acylhydrolase on chromosome 3, Aspartic proteinase-like and Thaumatin-like protein on chromosome 4, AT-hook DNA-binding and HSPRO2 on chromosome 6 as candidate genes for *P. thornei* resistance in the chickpea reference set. New sources of *P. thornei* resistant genotypes were identified that can be harnessed into breeding programs and putative candidate *P. thornei* resistant genes were identified that can be explored further to develop molecular markers and accelerate the incorporation of improved *P. thornei* resistance into elite chickpea cultivars.

## Introduction

Chickpea (*Cicer arietinum*) is an ancient legume that originated around the time when agriculture began during the Neolithic period in the Fertile Crescent (Abbo et al., 2003) in present day south-east Turkey and adjoining Syria. Domestication activities further led to diversification and adaptation of the crop to suit different environments. Presently, chickpea is an economically important crop, produced in more than 55 countries and ranks third in terms of area under harvest among legumes after soybean and dry beans (FAOSTAT, 2021). It is a climate resilient crop, cultivated worldwide due to its adaptation to arid and semi-arid regions. It is a valuable legume due to its ability to fix atmospheric nitrogen and also to increase biodiversity in cropping systems with cereals when used in rotation to break disease cycles (Mehta, 2014). The root-lesion nematode *Pratylenchus thornei* is an important pathogen of chickpea worldwide (Zwart et al., 2019). *Pratylenchus thornei*, using its stylet, a needle like hollow structure, pierces the root cells, enters in, feeds, and releases enzymatic secretions inside the plant cells. It is a migratory endoparasitic nematode and hence can traverse within and outside the root tissue intermittently. Upon infection, *Pratylenchus* spp. can complete an entire life cycle within the roots. About 4-5 generations of *Pratylenchus* spp. are completed in a growing season, leading to heavy build-up of the population density, which damages the root tissue and thereby limits the nutrient access to the plant (Fosu-Nyarko and Jones, 2016). As the severity of *P. thornei* infection increases, plants become prone to secondary infections by soil-borne fungal pathogens including *Fusarium oxysporum* f. sp. *ciceris* causing Fusarium wilt (Castillo et al., 1998) and *Rhizoctonia bataticola* causing dry root rot (Ali, 1997). Economic losses due to *P. thornei* infestation of up to 30% and 25% have been reported for the world’s largest chickpea producing countries India and Australia, respectively (Ali, 1997; Reen et al., 2014).

Among nematode management practices, breeding for resistance is the most sustainable approach, which is receiving more attention due to increasing climate change and environmental concerns associated with the use of chemical nematicides (Li et al., 2015). The pre-requisite for breeding for disease resistance is the availability of suitable genetic resistance. In the case of chickpea, there are limited sources of *P. thornei* resistance that have been identified to date, for example; in a study reported by Thompson et al. (2011), out of 182 chickpea accessions from the International Crops Research Institute for the Semi-Arid Tropics (ICRISAT), 16 accessions (including 5 wild *Cicer* spp.) showed resistance or moderate resistance towards *P. thornei*, with landrace accession ICC11323 showing the lowest population density of *P. thornei*. Reen et al. (2019) reported accessions of wild *Cicer* species *C. reticulatum* and *C. echinospermum* with improved levels of *P. thornei* resistance than previously identified by Thompson et al. (2011). Quantitative trait loci for *P. thornei* resistance have been mapped on Ca7 and Ca4 of *C. arietinum* in a bi-parental population (Khoo et al., 2021). The limited sources of *P. thornei* resistance in *C. arietinum* are partly due to the narrow genetic base of chickpea as a result of the domestication bottleneck (Abbo et al., 2003; Spillane and Gepts, 2000) and genetic drift (Cooper et al., 2000).

In such a scenario, plant genetic resources are an important pool from which novel sources of disease resistance can be identified. The largest collections of chickpea germplasm are held in genebanks at ICRISAT and the International Center for Agricultural Research in the Dry Areas (ICARDA) (Smýkal et al., 2015). In 2001, a core subset consisting of 1,956 chickpea accessions from the total 16,991 accessions in the ICRISAT germplasm collection was developed to capture the genetic diversity of the collection and enhance ultilisation of the germplasm resources for mining traits of interest in chickpea improvement programs (Upadhyaya et al., 2001). Later, a global composite collection of 3,000 chickpea accessions was developed, which consisted of the core subset from ICRISAT and additional accessions selected from the ICARDA genebank based on diversity in phenotypic traits and agroclimatological data linked to geographic origin (Upadhyaya et al., 2006). From the global composite collection, a reference set of 300 accessions, which captured 78% of the genetic diversity in the composite collection, was selected (Upadhyaya et al., 2008). The chickpea reference set provides a practical number of genetically diverse accessions to screen for traits of interest. The predominance of germplasm from Asia in the ICRISAT genebank is reflected in the chickpea reference set, with 66% of the accessions originating from Asia, in particular India and Iran (Upadhyaya et al., 2008). Approximately 90% of accessions in the chickpea reference collection are landraces (Upadhyaya et al., 2008; Varshney et al., 2019). Landraces are primitive varieties with a high capacity to tolerate biotic and abiotic stresses, resulting in high stability and intermediate yield level under low input agricultural systems (Zeven, 1998). Landraces therefore harbor a spectrum of alleles, which if used in breeding programs can help develop agronomically superior cultivars with enhanced stress tolerance and disease resistance.

*P. thornei* resistance in chickpea is a polygenic trait (Channale et al., 2021) and genome-wide association study (GWAS) serves as an ideal method for genetic dissection of complex traits. GWAS takes advantage of evolutionary recombination events in crops to determine the molecular variations associated with the traits including disease responses (Nordborg and Weigel, 2008; Braulio and Sylvie, 2012). It has been widely used in different crop species to map genomic loci associated with economically important traits (Gangurde et al., 2022). In chickpea, genomic regions associated with Ascochyta blight (Li et al., 2017; Farahani et al., 2022; Raman et al., 2022), *Pythium ultimum* (Agarwal et al., 2022), nutrient traits (Diapari et al., 2014; Jadhav et al., 2015; Upadhyaya et al., 2016a; Upadhyaya et al., 2016b) and abiotic stress (Thudi et al., 2014) have been mapped using GWAS.

Single nucleotide polymorphism (SNP) markers are popular markers in GWAS due to their genome-wide abundance and flexibility for high- to ultra-high-throughput detection platforms, which enables genome-wide scanning, and fine mapping of target regions (Mammadov et al., 2012). With the advent of low-cost genotyping platforms, GWAS is a preferred method to dissect genetics of resistance in different crop species against different plant parasitic nematodes. To the best of our knowledge *P. thornei* resistance using GWAS has been reported only in common wheat (*Triticum aestivum* L.). Kumar et al. (2021) reported nine genes with putative roles in *P. thornei* resistance in common wheat using pan-Indian wheat germplasm. In addition, Dababat et al. (2016) and Sohail et al. (2022) reported nine and four markers, respectively, linked to *P. thornei* resistance in spring bread wheat collections held by CIMMYT (International Maize and Wheat Improvement Center). Among other plant parasitic nematode species, marker-trait associations have been identified using GWAS for resistance to root-knot nematode (*Meloidogyne javanica)* resistance in soybean (Alekcevetch et al., 2021), root-knot nematode (*M. graminicola)* resistance in Indian wild rice accessions (Hada et al., 2020), and cyst nematode (*Heterodera filipjevi*) in wheat (Pariyar et al., 2016).

The above-mentioned studies were performed with single-locus and multi-locus models using the Bonferroni correction method. Bonferroni correction is a conservative approach that may result in many important associations being eliminated because they fail to satisfy the stringent criteria of significant tests for complex traits in crops (Wang et al., 2016). Recent multi-locus model methods for GWAS do not require Bonferroni correction and instead use LOD = 3.0 (or P = 0.0002) as a significance cutoff to balance the high power and low false positive rate to detect significant associations (Zhang et al., 2019). Several multi-locus GWAS models have been developed, including multi-locus random-SNP-effect mixed linear model (mrMLM) (Wang et al., 2016), fast multi-locus random-SNP-effect efficient mixed model analysis (FASTmrEMMA) (Wen et al., 2018; Wen et al., 2020), and Iterative modified-sure independence screening expectation-maximization-Bayesian least absolute shrinkage and selection operator (ISIS EM-BLASSO), (Tamba et al., 2017). These multi-locus GWAS models have been shown to have increased statistical power over single locus GWAS methods for the detection of quantitative trait nucleotides (QTNs) of small effect associated with complex traits. For example, candidate genes related to drought stress in cotton (*Gossypium hirsutum* L.) were identified using a multi-locus method GWAS (Hou et al., 2018). Also, salt tolerant loci were identified in rice (*Oryza sativa* L.) by using a multi-GWAS approach (Cui et al., 2018).

We evaluated the chickpea reference set (Upadhyaya et al., 2008) for *P. thornei* resistance in glasshouse experiments in each of two years and identified chickpea lines resistant to *P. thornei*. Since the chickpea reference set is a global genetic resource that has been phenotyped for different traits, it is of interest to identify accessions that carry multiple disease resistance or other traits of interest. We used reports in the literature to identify accessions in the chickpea reference set with combined resistance to *P. thornei* and other biotic as well as abiotic stresses. We tested six multi-locus GWAS models to understand the effectiveness of the models and identified robust QTNs associated with *P. thornei* resistance. Candidate genes for *P. thornei* resistance were further identified. To the best of our knowledge, this is the first genome-wide association study for *P. thornei* resistance in chickpea.

## Materials and methods

### Plant materials

The association panel consisted of a total of 278 chickpea accessions: 276 accessions of the chickpea reference set (Upadhyaya et al., 2006), originating from 32 countries, obtained from the Australian Grains Genebank, Horsham, Victoria, Australia, plus two Australian chickpea varieties (PBA HatTrick and PBA Pistol) (**Supplementary Table 1**). An additional seven putatively *P*. *thornei* resistant *C. arietinum* accessions (**Supplementary Table 2**) identified from previous studies were also evaluated (Castillo et al., 2008; Thompson et al., 2011; Rodda et al., 2016), for *P. thornei* resistance. Well characterized chickpea (9) and wheat (8) lines, covering the range of responses to *P. thornei* from resistant to very susceptible, were included in the phenotyping experiments as check cultivars. The chickpea check cultivars were Sonali, ILWC 123, ILWC 184, PBA Boundary, Flipper, Yorker, Sona, Kyabra, PBA Seamer. The wheat check cultivars were CP1133872, GS50a, Gauntlet, QT8447, Sunzell, EGA Kidman, Sunguard and Strezlecki. All the accessions were phenotyped twice except for 27 chickpea reference set accessions and three accessions identified as sources of resistance, as they were unavailable at the time of Experiment 1. Unplanted pots inoculated with *P. thornei* were also included as negative controls.

### Glasshouse and plant growth conditions

The association panel and check genotypes were evaluated for *P. thornei* resistance in inoculated glasshouse pot experiments. Treatments consisted of a single pot (containing one plant of a genotype). Treatments were randomly arranged on glasshouse benches according to a row-column design with three replicates of each treatment. A replicate was equal to one block and each block was allocated to a separate adjacent glasshouse bench. The glasshouse experiment was conducted twice, with Experiment 1 conducted at the Leslie Research Facility, Toowoomba from July to November 2018, and Experiment 2 conducted in a new glasshouse with improved air temperature control at the University of Southern Queensland, Toowoomba from July to November 2020.

All the glasshouse benches were set up with a self-regulating bottom watering system with water flow regulated by a float valve set to a water tension of 20 mm. Capillary matting (Bidim^®^; Geofabrics Australasia Pty Ltd) was placed over each bench with its edges immersed in the water troughs attached to the bench. Square pots with dimension 70-mm square, 150-mm high, were filled with 330 g (oven-dry equivalent) of a black earth of the Waco clay soil association (Beckmann and Thompson, 1960) pasteurised with aerated steam at 85°C for 30 min (modified from Thompson, 1990). Initially, 80% (252 g) of the total soil was mixed with 30 mL of nutrient solution, to provide 200 mg NO_3_-N, 25 mg P, 88 mg K, 36 mg Na, 285 mg Ca and 5 mg Zn/kg soil and pots were filled. The pots were placed on the pre-wetted benches and flooded with water to 10-20 mm depth to ensure that capillary action between soil and matting was established. The pots were left overnight to allow the soil to take up water before planting and inoculation. For planting, three seeds of each accession were placed on top of the moist soil and inoculated with 1 mL of commercially available rhizobium culture (Group N, isolate CC1192 [*Mesorhizobium ciceri*]) prior to inoculation with 3,300 *P. thornei* (equivalent to 10,000 nematodes/kg oven-dry soil) suspended in 10 mL of water. The remaining 20% (78 g) of soil was placed over the seeds and nematode inoculum as a cap. One week after emergence, plants were thinned to one plant per pot. The extra plants were removed carefully by cutting off the shoot below the crown with a scalpel, leaving the roots in the pot. Air and soil temperatures were maintained between 20 and 25 °C, the optimum temperature for *P. thornei* reproduction (Thompson et al., 2015), using under-bench heating, shade cloth (as required), evaporative coolers (Experiment 1) and air conditioning (Experiment 2).

### Plant harvest, nematode extraction, and enumeration

The plants were grown for 18 weeks. Before harvest, the water supply was turned off to allow the soil to dry down to ~45% moisture content for effective soil sampling and processing for nematode extraction. Plant maturity on the BBCH scale (Lancashire et al., 1991), tiller number, and plant height were recorded at harvest. The plant shoots were cut off at the base just above the soil level, oven dried at 80°C for 48 h and shoot dry biomass was recorded. The pots with soil were packed in polythene bags and stored at 4°C until further processing. The soil and roots from each pot were thoroughly mixed and the roots were cut into approximate 10 mm lengths. The mixture was subsampled with 100 g for determination of gravimetric moisture content and 150 g for nematode extraction. For gravimetric moisture content, the soil mixture was dried at 105°C for 48 h. The nematodes were extracted by the Whitehead tray method (Whitehead and Hemming, 1965). The nematodes were collected on a 20-µm sieve, concentrated in approximately 15 mL water, which was poured into a 30 mL vial and stored at 4°C. *Pratylenchus thornei* populations extracted from soil and roots were counted under a compound microscope (4x Olympus BX53) using a Peters counting chamber of 1 mL capacity (Chalex Corporation, Portland, Oregon, USA).

### Statistical analyses

Due to heterogeneity of variances as assessed by the Shapiro Wilk test for normality, *P. thornei* counts were transformed by log_e_ (x + 1), where x = number of *P. thornei*/kg of soil and roots (oven dry equivalent). Pearson’s correlation coefficient was used to determine linear correlation between log_e_(*P. thornei*/kg +1) of accessions in Experiment 1 and Experiment 2 in GraphPad Prism (2022). Experiment 1 and Experiment 2 were analyzed together using a linear mixed model (LMM) to obtain best linear unbiased estimates (BLUE) values with the following terms included in the model: genotype as a fixed effect and replication nested in experiment as random effect. Multiple comparisons for REML was applied to obtain Fishers protected least significant differences (l.s.d.) (at 5% significance level). The analyses were performed using Genstat v21 (VSN International, 2020). The range of log_e_ (*P. thornei/kg+*1) as BLUE values was subdivided into eight equal subranges based on the procedure of Thompson et al. (2020). These subranges were assigned the following ordinal alpha categories, resistant (R), resistant to moderately resistant (R-MR), moderately resistant (MR), moderately resistant to moderately susceptible (MR-MS), moderately susceptible (MS), moderately susceptible to susceptible (MS-S), susceptible (S), susceptible to very susceptible (S-VS) with calibration to this S-VS category based on the wheat cultivar Strzelecki (Thompson et al., 2020). A histogram of frequencies of accessions in these eight classes was produced.

### Identification of *P. thornei* resistant chickpea accessions with resistance to other biotic and/or abiotic stresses

As the chickpea reference collection is a critical resource for studying genetic diversity, these accessions have been phenotyped for various traits by numerous researchers. The chickpea accessions from different phenotyping studies previously reported as R or, MR to the biotic stresses Ascochyta blight, Botrytis grey mould, dry root rot, and Fusarium wilt (Pande et al., 2006), or abiotic stresses (Serraj et al., 2004), or accessions with superior agronomic characteristics (Meena et al., 2010) were compared with the accessions in the current study falling within the categories of R, R-MR, or MR to *P. thornei*.

### Genotyping and population stratification

The SNP dataset for the chickpea reference set germplasm collection was selected for the study as per Varshney et al. (2019). Briefly, the SNP data set for 429 accessions (Varshney et al., 2019) was sorted using the sort tool in the Galaxy Australia web platform at usegalaxy.org.au (Galaxy Community, 2022) to obtain SNP data of the 278 accessions of the association panel. The additional chickpea accessions phenotyped in this study were not included in the 429 accessions genotyped by Varshney et al. (2019) and hence were omitted from the GWAS analysis. The SNP dataset for the 278 chickpea accessions was filtered for missing values (≥20%) and minor-allele frequency (MAF < 5%) using Trait Analysis by aSSociation, Evolution and Linkage (TASSEL) 5.0 (Glaubitz et al., 2014) and the Variant Call Format (VCF) filter tool in Galaxy Australia. Population stratification of the reference set was identified using ADMIXTURE software (version 1.3) (Liu et al., 2020) to measure the proportion of individual ancestry and sub-populations (K). Since the ADMIXTURE algorithm is based on independent loci, pruning of the dataset based on linkage disequilibrium (LD) was carried out using PLINK1.9 (Chang, 2015) command with default parameters. The cross-validation (CV) error was tested for K=1 to 20 for the best value of K. Visual inspection of ADMIXTURE plots generated using the pong tool (Behr et al., 2016) was also used to decide the best K value.

### Genome-wide association and candidate gene analysis

For GWAS, six multi-locus models, namely, mrMLM (Wang et al., 2016), FASTmrMLM, (Tamba and Zhang, 2018), FASTmrEMMA (Wen et al., 2018; Wen et al., 2020), pKWmEB (Ren et al., 2018), pLARmEB (Zhang et al., 2017), ISIS EM-BLASSO (Tamba et al., 2017) were implemented using the mrMLM R package (Zhang et al., 2020) with all the default parameters. The threshold of LOD score for significant QTN was set at 3.0 for all the six methods. The QTNs were considered reliable if they were identified by two or more models. The flanking regions of these QTNs were analysed with the open source computational platform JBrowse2 (Diesh et al., 2022) using track Cicer arietinum CDCFrontier.gnm3.ann1.NPD7.gene_models_main.gff3 (https://cicer.legumeinfo.org). Candidate genes were defined as annotated genes within 315 kb either side of a QTN, based on the LD decay of the chickpea reference set reported by Varshney et al. (2019). The candidate genes were verified for their function based on the literature reporting roles associated with plant-pathogen interactions.

## Results

### Phenotypic evaluation of *Pratylenchus thornei* resistance

**Figure 1:**
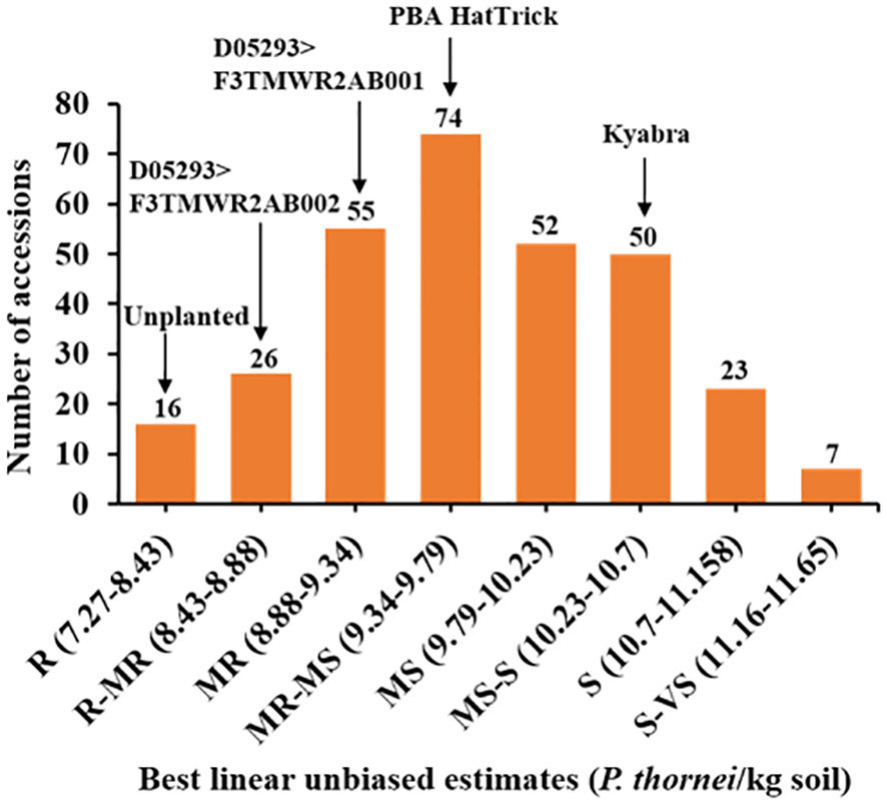
Frequency distribution histogram of the chickpea reference collection and additional resistance sources (total 303 accessions) for *P. thornei* population densities. Data shown is the best linear unbiased estimate (BLUE) values for combined analysis of Experiment 1 and Experiment 2. Resistant **(R)**, resistant to moderately resistant (R-MR), moderately resistant (MR), moderately resistant to moderately susceptible (MR-MS), moderately susceptible (MS), moderately susceptible to susceptible (MS-S), susceptible **(S)**, susceptible to very susceptible (S-VS).

Pearson’s correlation coefficient indicated that Experiment 1 and Experiment 2, analysed for log_e_ (*P. thornei*/kg*+*1) of soil, were moderately correlated (r = 0.3, P < 0.0001, n=264) (**Supplementary Figure 1**). In order to obtain the most representative phenotypic values, LMM analysis of the combined data sets was used to obtain BLUEs (**Supplementary Tables 3, 4**). *Pratylenchus thornei* population densities were normally distributed (Shapiro Wilk test = 0.997; P = 0.789) (**Figure 1**). Approximately, 5% of the total accessions belonged to the R category (**Supplementary Table 5**), 9% of the total accessions belonged to R-MR category and 18% of accessions to the MR category. The lowest percentage of accessions (2%) belonged to S-VS category. The well characterized wild relative *C. echinospermum* ILWC 123, used as a resistant check cultivar had *P. thornei* population density of 2,660 *P. thornei*/kg of soil. The only two other wild relatives tested in the study, ICC 20190 and ICC 20183, belonged to R and R-MR categories with *P. thornei* population densities of 2,012 *P. thornei*/kg of soil and 6,039 *P. thornei*/kg of soil, respectively. Chickpea breeding line D05253>F3TMWR2AB001 was in the R-MR category with *P. thornei* population density of 5,340 *P. thornei*/kg of soil, and D05293>F3TMWR2AB002 was in MR category with 8,955 *P. thornei*/kg of soil. MR check cultivar PBA HatTrick had high population density with 16,498 *P. thornei*/kg of soil and MS-S check cultivar Kyabra produced high *P. thornei* density of 35,846/kg of soil. Eight of the resistant accessions produced significantly lower *P. thornei* population densities than the Australian commercial cultivar PBA HatTrick. The wheat check cultivars used in the study to confirm the *P. thornei* multiplication performed as expected.

### Identification of *P. thornei* resistant chickpea accessions with resistance to other biotic and/or abiotic stresses

The chickpea accessions in the present study in R and R-MR ratings for resistance to *P. thornei* were compared with reports in the literature where the chickpea reference set has been phenotyped for other biotic and abiotic traits (**Table 1**). ICC 15697 was resistant to *P. thornei*, moderately resistant to Botrytis grey mould and tolerant to salinity. ICC 8950, ICC 5135, ICC 6816, ICC 95, ICC 14831 were resistant to *P. thornei* and moderately resistant to Fusarium wilt. ICC 8058 was resistant to *P. thornei* and Fusarium wilt ICC 12328 was moderately resistant to *P. thornei* and moderately resistant to Botrytis grey mould, Ascochyta blight, and dry root rot and with superior agronomic traits of grain yield and seed size.

**Table 1:** The chickpea accessions in resistant (R) and moderately resistant (MR) categories to *Pratylenchus thornei* of present study collated with reports of multiple disease resistance or tolerance to abiotic stress and/or with superior agronomic traits from the literature.

Accession	Pt	FW	FW_a_	BGM	AB	DRR	Abiotic stress/Agronomic traits
**ICC 8950**	R	MR	…	…	…	…	…
**ICC 8058**	R	…	R	…	…	…	…
**ICC 5135**	R	MR	…	…	…	…	…
**ICC 6816**	R	MR	…	…	…	…	…
**ICC 15697**	R	…	…	MR	…	…	Salinity
**ICC 95**	R	MR	…	…	…	…	…
**ICC 14831**	R	MR	…	…	…	…	…
**ICC 12328**	MR	…	…	MR	MR	R	Yield and seed size

AB, Ascochyta blight; BGM, Botrytis grey mould; DRR, dry root rot; FW, Fusarium wilt; FW_a_, Fusarium wilt asymptomatic; MR, moderately resistant; R, resistant; Pt, *Pratylechus thornei*.

### Genotyping and population stratification

**Figure 2:**
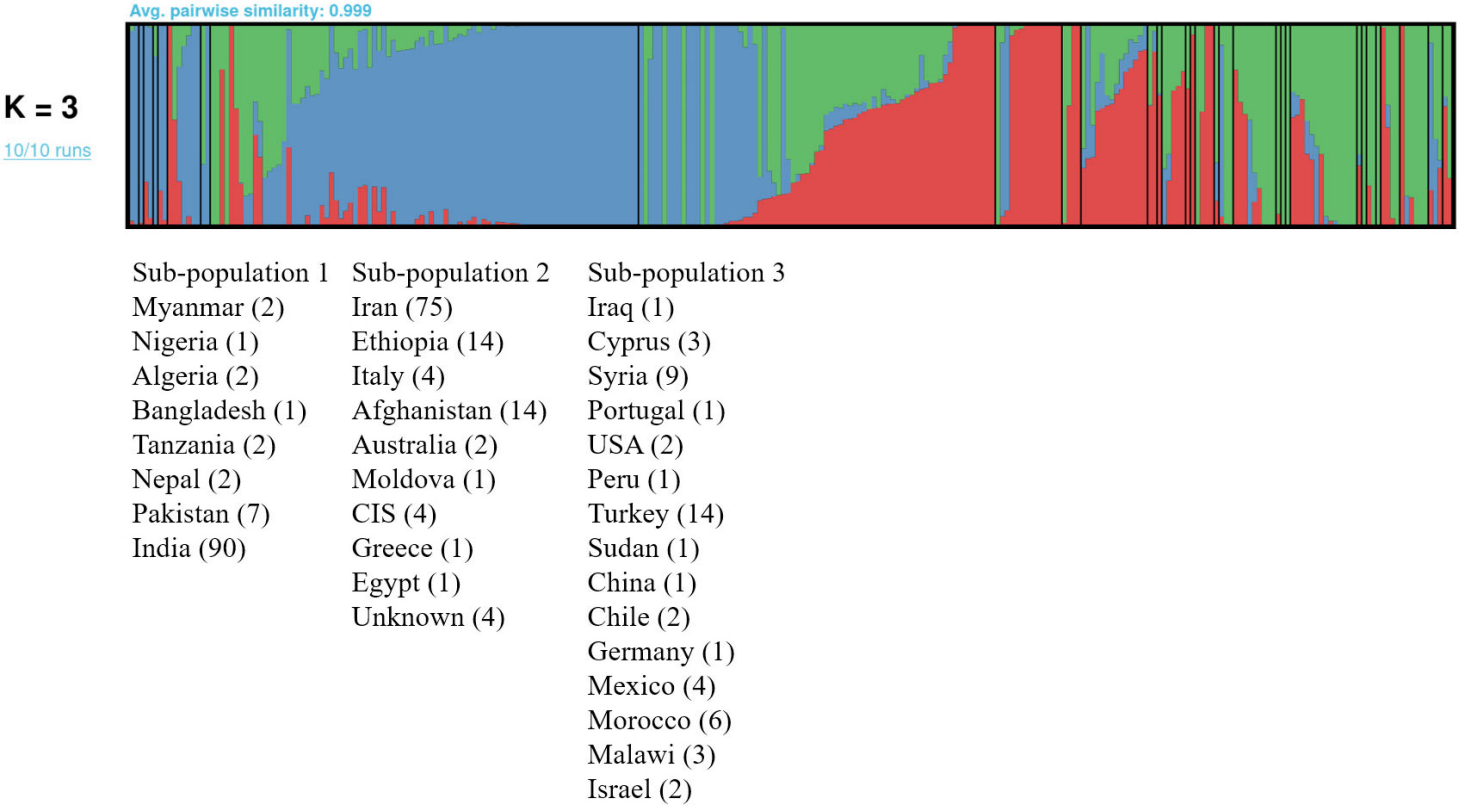
ADMIXTURE analysis of the chickpea association panel of 278 accessions using 112,998 SNPs and the pong tool formed 3 sub-populations: sub-population 1 (Blue), sub-population 2 (Red) and sub-population 3 (Green). The accessions in sub-populations were labelled as per country of origin, with the number of accessions in parentheses. The black vertical lines separate the countries within each sub-population. All the accessions in the respective sub-populations shared more than 50% ancestry except for accessions from Iraq, which had similar ancestry in all sub-populations and that of CIS and Iraq with ancestry between 40 to 50% for respective sub-populations.

Pruning LD-wise the chickpea association panel of 278 accessions resulted in 112,998 SNPs that were used to assess population stratification. ADMIXTURE is based on maximum likelihood to estimate allele frequencies in each group or sub-population and assign each individual ancestry to one or more of these group or sub-populations. The ADMIXTURE run depicted continuous decline after K=3 (**Supplementary Figure 2**). The best K value (K=3) was confirmed by visual inspection of the plot generated (**Figure 2**). The sub-population 1 comprised 107 accessions with most accessions in the group originating from India. The sub-population 2 comprised 120 accessions with the majority of the accessions originating from Iran, whereas sub-population 3 comprised the least number of accessions (n=51) with most accessions originating from Turkey.

### Genome-wide association and candidate gene analysis

**Figure 3:**
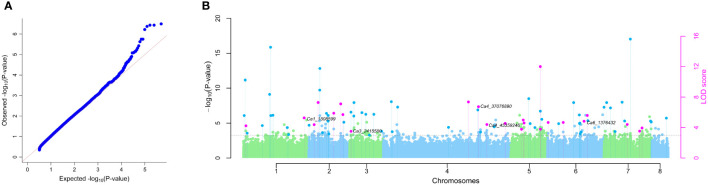
Multi-locus GWAS. **(A)** Quantile-quantile plot for comparison between expected and observed –log10 (P-values). **(B)** Manhattan plot for significant QTNs associated with *P. thornei* resistance in chickpea using mrMLM, FASTmrMLM, FASTmrEMMA, pKWmEB, pLARmEB and ISIS EM-BLASSO models. The -log10 (P-value) and the LOD score of SNP across the genome (y-axes) were plotted against their respective position on each chromosome (x-axis). The pink dots above dotted vertical lines represent all the QTNs commonly identified by multiple approaches and the dark blue dots above dotted vertical lines indicate QTNs identified by a single GWAS model. Candidate gene analysis revealed QTNs Ca1_1808599, Ca4_37076890 and Ca6_1376432 were flanked by Receptor-linked kinases (RLKs). QTN Ca3_2415580 was flanked by GDSL-like Lipase/Acylhydrolase. QTN Ca4_45359246 was flanked by Aspartic proteinase like and Thaumatin-like protein and QTN Ca6_1376432 was flanked by AT-hook DNA-binding and HSPRO2.

The SNP data of the chickpea association panel after filtration for missing values (≥20%) and minor-allele frequency (MAF < 5%) resulted in 492,849 SNPs for further analysis. The number of SNPs per chromosome were 74,764 (Ca1), 47,430 (Ca2), 39,254 (Ca3), 14,7829 (Ca4), 42,616 (Ca5), 65,589 (Ca6), 55,798 (Ca7), and 18,569 (Ca8) (**Supplementary Figure 3**). GWAS using six different models in the mrMLM package resulted in detection of 24 QTNs (**Table 2** and **Figure 3**) distributed on all of the chickpea chromosomes, except for chromosome 8. Ten QTNs were detected by three to five of the six multi-locus GWAS models, with an additional 14 QTNs detected by two models. Among the six multi-locus GWAS models FASTmrMLM detected the highest number of QTNs (19), followed by pKWMEB (16) and ISIS EM-BLASSO (12). The other multi-locus GWAS models detected five or fewer QTNs each. The FASTmrMLM model outperformed other models, co-detecting 14 of the 16 QTNs detected by pKWMEB, and 10 of the 12 QTNs detected by ISIS EM-BLASSO. Mining the results of several multi-locus GWAS has been recommended to improve the detection power and robustness of GWAS (Zhang et al., 2019), particularly for QTNs with small allelic effects (Wang et al., 2016).

**Table 2:** The significant quantitative trait nucleotides (QTNs) for *Pratylenchus thornei* resistance or susceptibility in chickpea detected by two or more multi-locus GWAS methods.

QTN	Maker position (bp)	Chromosome	QTN effect^a^	LOD score	r^2^ (%)^b^	MAF	Method^c^	Allele that was coded as 1	No. of genes flanking the QTN
**Ca1_1808599**	1808599	1	-0.109	4.232	2.986	0.278	2,4	AA	15
**Ca2_32257204**	32257204	2	-0.111	7.109	1.530	0.365	1,5	AA	3
**Ca3_2415580**	2415580	3	-0.162	3.503	2.584	0.058	2,4	GG	13
**Ca4_45359246**	45359246	4	-0.138	4.548	1.176	0.151	5,6	TT	17
**Ca4_37076890**	37076890	4	-0.262	7.366	2.405	0.179	1,3	AA	12
**Ca5_17048541**	17048541	5	-0.172	4.599	4.369	0.107	4,6	AA	0
**Ca5_15873614**	15873614	5	-0.184	4.987	4.341	0.109	2,4	AA	0
**Ca5_36962566**	36962566	5	-0.231	12.262	5.293	0.200	2,4,5,6	CC	4
**Ca6_1376432**	1376432	6	-0.144	4.056	2.762	0.207	2,4,5	GG	25
**Ca6_38461529**	38461529	6	-0.147	4.831	3.311	0.250	1,2	GG	6
**Ca6_43054424**	43054424	6	-0.190	6.077	5.313	0.374	2,4,6	GG	8
**Ca7_30958644**	30958644	7	-0.180	5.027	5.163	0.254	2,4,6	TT	13
**Ca7_36076912**	36076912	7	-0.125	5.202	3.111	0.284	1,2,4	GG	11
**Ca1_41591258**	41591258	1	0.308	5.269	4.353	0.255	3,4	TT	7
**Ca2_10135999**	10135999	2	0.145	6.983	2.965	0.208	2,4,6	TT	13
**Ca2_33400846**	33400846	2	0.195	5.705	5.015	0.142	2,4	GG	6
**Ca2_4843786**	4843786	2	0.196	4.506	3.649	0.070	2,4,6	TT	12
**Ca2_31149419**	31149419	2	0.277	6.800	3.157	0.421	2,3,4,6	CC	0
**Ca4_38232015**	38232015	4	0.134	6.739	2.505	0.229	1,2	TT	11
**Ca4_39047069**	39047069	4	0.188	4.402	3.156	0.088	2,6	AA	11
**Ca5_37270329**	37270329	5	0.168	3.784	1.949	0.076	2,6	TT	5
**Ca5_11929738**	11929738	5	0.208	3.908	3.229	0.086	2,5,6	TT	12
**Ca6_10986884**	10986884	6	0.112	3.925	2.063	0.138	2,4,6	CC	12
**Ca7_34451716**	34451716	7	0.116	3.526	2.433	0.162	2,4	GG	8

a Positive QTN effect indicates association with increased *P. thornei* resistance; negative QTN effect indicates association with increased *P. thornei* resistance.

b r^2^ (%), phenotypic variation of traits explained by each QTN.

c GWAS models indicated as 1 (mrMLM), 2 (FASTmrMLM), 3 (FASTmrEMMA), 4 (pKWmEB), 5 (pLARmEB) and 6 (ISIS EM-BLASSO).

The effect size relates to an increase or a decrease in value of the trait under study, meaning a positive effect relates to higher nematode population density and a negative effect relates to lower nematode population density. Thirteen QTNs were associated with lower nematode population density, indicating increased resistance, and 11 QTNs were associated with higher nematode population density, indicating increased susceptibility.

Analysis of single experiment data identified 23 QTNs (6 associated with decreased *P. thornei* population densities and 17 associated with increased *P. thornei* population densities) for Experiment 1 (**Supplementary Table 6**). One of these QTNs (Ca2_31149419) was detected as a significant QTN using BLUEs from combined Experiments 1 and 2 (**Supplementary Table 7**). Analysis of Experiment 2 data revealed 17 QTNs (10 associated with decreased *P. thornei* population densities and 7 associated with increased *P. thornei* population densities). Eight of the QTN detected in the Experiment 2 dataset were in common with analysis of the combined dataset (**Supplementary Table 7**).

Of the 24 significant QTN positions identified by analysis of the combined dataset, 20 QTNs were flanked by annotated genes (**Supplementary Table 8**). These candidate genes were analysed for their putative role in *P. thornei* resistance based on reported functions in biotic and abiotic stress responses in the literature. A total of six candidate genes were identified with previously reported roles in plant-pathogen interactions. Receptor-linked kinases (RLKs), a common multigene family, were located on chromosomes 1, 4 and 6 flanking QTNs Ca1_1808599, Ca4_37076890 and Ca6_1376432, respectively; GDSL-like Lipase/Acylhydrolase was identified on chromosome 3 flanking QTN Ca3_2415580; Aspartic proteinase like and Thaumatin-like protein were identified on chromosome 4 flanking QTN Ca4_45359246; and AT-hook DNA-binding and HSPRO2 were identified on chromosome 6 flanking QTN Ca6_1376432 (**Figure 3**).

## Discussion

A wide variation of resistance and susceptibility to *P. thornei* was observed among the accessions of the chickpea reference collection. Fourteen accessions were classified as resistant to *P. thornei*, of which 11 accessions were landraces, two were breeding lines and one a wild relative (*C. echinospermum*). While wild relatives of chickpea (*C. echinospermum* and *C. reticulatum*) have been identified with improved levels of *P. thornei* resistance over current commercial chickpea cultivars grown in Australia (Thompson et al., 2011; Reen et al., 2019), sources of *P. thornei* resistance in the *C. arietinum* cultigen are favoured by plant breeders over the need to create interspecific crosses, as fewer backcrosses are required to introgress resistance while recovering an elite background. Thus, these landrace accessions with *P. thornei* resistance identified in this study constitute a valuable resource for increasing resistance to *P. thornei* in chickpea cultivars in Australia and globally.

With limited reports on the mechanisms of resistance against migratory endoparasitic nematodes (Mathew and Opperman, 2020), all annotated genes flanking the QTN were further investigated, with those identified with a reported role in biotic or abiotic stress considered candidate genes for *P. thornei*. However, all genes in the flanking regions of the significant QTN should be considered for further investigation through differential expression and functional analysis.

Among genes flanking the QTNs, Ca1_1808599, Ca4_37076890 and Ca6_1376432 on chromosomes 1, 4 and 6, respectively, the receptor-linked kinases (RLKs) subtype - lectin RLKs (LecRLKs), was found to be the common multi-gene family. The importance of receptor-like kinases in resistance to *P. thornei* have been reported in a fine mapping study of *P. thornei* resistance loci in wheat (Rahman et al., 2020) and overexpression of receptor-like kinase upon *P. thornei* infection was reported in resistant chickpea genotypes (Channale et al., 2021). LecRLKs act as sensors and mediators of signals from the plant cell surface to intracellular compartments, playing crucial roles in plant development and responses to abiotic and biotic stresses (Sun et al., 2020). In a study by Cheng et al. (2013), OslecRK (*Oryzae sativa* lectin receptor-like kinase) was found to be a pleiotropic gene involved not only in mounting immune response against biotic stress but also in the germination of seeds.

For Ca3_2415580, a GDSL-like Lipase/Acylhydrolase superfamily protein gene was located in the upstream region of the QTN. GDSL-type esterase/lipase protein is a hydrolytic enzyme that possesses broad substrate specificity to compounds such as thioesters, aryl esters, phospholipids and amino acids (Akoh et al., 2004). The GDSL family is involved in a myriad of roles including plant growth and development, secondary metabolism, plant immunity, and biotic and abiotic stresses (Su et al., 2020). GDSL lipase was demonstrated to have constitutive expression in resistant cultivars of sweet potato (*Ipomoea batatas*) upon infection with root-knot nematode *Meloidogyne incognita*. Enhanced expression of GLIP1 (GDSL LIPASE-LIKE 1) increased resistance of *Arabidopsis thaliana* to *Alternaria brassicicola*, *Erwinia carotovora* and *Pseudomonas syringae* (Kwon et al., 2009).

The genes flanking QTN Ca4_45359246 on chromosome 4 included aspartic proteinase-like, its isoform, and thaumatin-like proteins. Aspartic proteinases are a widely distributed class of proteinases in plants involved in an array of biological processes including plant immunity (Figueiredo et al., 2021). They are also involved in regulating the degradation of proteins induced during stress such as pathogenesis-related proteins like thaumatin to prevent their overaccumulation. They are also known to hydrolyze proteins secreted by invasive pathogens (Schaller and Ryan, 1996). Thaumatin-like protein is a multi-gene family also involved in various functions in host-pathogen interaction like biotic stress, abiotic stress tolerance, and cell signalling (Liu et al., 2010). The role of thaumatin-like protein in *P. thornei* resistance can also be corroborated from the fact that thaumatin-like protein was overexpressed in a resistant chickpea cultivar on exposure to *P. thornei* (Channale et al., 2021).

The genes flanking QTN Ca6_1376432 involved in pathogen defence and plant development included Predicted AT-hook DNA-binding and nematode resistance protein-like HSPRO2-like genes. The AT-hook gene family is highly conserved across all land plants being involved in plant growth and development, response to stress and hormonal stimulus (Zhao et al., 2014; Wang et al., 2021). In one of the studies reported by Kim et al. (2007), the AT-hook DNA-binding protein-encoding gene CaATL1 was specifically induced upon pathogen inoculation. Transgenic tomato plants overexpressing CaATL1 showed enhanced resistance against *Pseudomonas syringae* and *Phytophthora capsici* (Kim et al., 2007). The HSPRO2-like gene was demonstrated to be a positive regulator of basal resistance, which functions downstream of the salicylic acid pathway in *Arabidopsis* against *P. syringae* (Murray et al., 2007). Also, three putative resistance genes were identified encoding nematode resistance HSPRO2-like proteins in response to *Pseudomonas syringae pv syringae* in *Medicago sativa* (Nemchinov et al., 2017).

Among candidate genes flanking QTN Ca3_15978889, detected by multiple GWAS methods in Experiment 2, was Bowman-Birk trypsin inhibitor. Bowman-Birk trypsin inhibitor is one of types of serine proteinase inhibitors (PIs) rich in legume seeds (Srinivasan et al., 2005) exhibiting strong inhibitory activity against trypsin, chymotrypsin or both. It plays a major role in plant defence due to its anti-digestive properties (Murdock and Shade, 2002; Ferry et al., 2004; Zavala et al., 2004). In a study reported by Rashed et al., 2008, transcripts with Bowman-Birk trypsin inhibitor were overexpressed in soybean in response to soybean cyst nematode, *Heterodera glycines.*

Similarly, candidate genes flanking two QTNs on chromosome 7, Ca7_15462252 and Ca7_4192522 identified by combined GWAS analysis and in Experiment 2 included salicylic acid carboxyl methyltransferase and glutathione S-transferase family protein. Salicylic acid carboxyl methyltransferase is synthesized in plants from salicylic acid and plays important roles in defence against microbial and insect pests (Liu et al., 2010). Salicylic acid dependent immune priming of defense genes was reported in chickpea resistant genotype upon interaction with *P. thornei* (Channale et al., 2021). Glutathione S transferases are multifunctional enzyme and are highly induced in stress conditions and are also known to be receptors of salicylic acid (Gullner et al., 2018).

The chickpea reference set is a valuable collection capturing the diversity in landrace chickpea germplasm that has been phenotyped by different research groups in glasshouse and field trials for various biotic stresses, including Ascochyta blight, Fusarium wilt, Botrytis grey mould, dry root rot, abiotic stresses including heat, drought, and salinity, and for agronomic traits like yield and seed weight. Accessions previously reported as resistant (ICC 6816, ICC 95, ICC 14831) or asymptomatic (ICC 8058) to Fusarium wilt (Pande et al., 2006) were identified in our study to be resistant to *P. thornei*. Accessions with dual resistance will be promising sources for chickpea breeding programs to produce superior commercial cultivars for regions where *F. oxysporum* and *P. thornei* are known to co-infect chickpea e.g., Southern Spain (Castillo et al., 1998). Similarly, ICC 15697 was resistant to *P. thornei* in this study and has been reported to be moderately resistant to Botrytis grey mould (Pande et al., 2006) and tolerant to salinity (100mM) (Serraj et al., 2004). Along with multiple disease resistance, it is important that grain yield is also maintained, yet, building a disease-resistant and high-yielding crop is often a major challenge to breeders and scientists (Wang and Dong, 2021). Notably, we identified the high yielding accession ICC 12328 (Meena et al., 2010) as moderately resistant to *P. thornei*, and this accession was also reported as moderately resistant to Ascochyta blight, dry root rot, and Botrytis grey mould (Pande et al., 2006). *Pratylenchus thornei* reproduction was reported to be significantly increased in chickpea cultivars susceptible to *F. oxysporum* f. sp. *ciceris* (Castillo et al., 1998). Thus, the *P. thornei-*resistant landraces identified in this study will not only be invaluable for breeding resistant modern cultivars to protect the chickpea crop itself and for integrated management of *P. thornei* in cropping systems with cereal host crops, but can also be of value in breeding multiple-disease resistant chickpea cultivars.

## Conclusion

Six multi-locus GWAS models were used to detect reliable QTNs associated with *P. thornei* resistance in chickpea landraces. A total of 24 QTNs were co-detected by at least two different multi-locus GWAS models. Candidate gene analysis identified receptor-linked kinases (RLKs) on chromosome 1, 4 and 6, GDSL-like Lipase/Acylhydrolase on chromosome 3, Aspartic proteinase-like and Thaumatin-like protein on chromosome 4, AT-hook DNA-binding and HSPRO2 on chromosome 6 as candidate genes for *P. thornei* resistance. Following verification of these candidate genes using differential gene expression studies, cost-effective breeder-friendly markers can be developed to aid marker assisted selection. Such molecular tools will facilitate efficient introgression of *P. thornei* resistance into elite chickpea cultivars and provide important insights into the mechanisms of *P. thornei* resistance.

## Data availability statement

The original contributions presented in the study are included in the article/**Supplementary Material**, further inquiries can be directed to the corresponding authors.

## Author contributions

RZ, MT, and JT conceived the experiments. SC conducted the experiments. SC, RZ and MT contributed to data analysis and interpretation. JT and RV reviewed and edited the manuscript. SC drafted the manuscript with input from the other co-authors. All authors critically revised and approved the manuscript. All authors contributed to the article and approved the submitted version.
