# Supplementary material for: Multi-locus genome-wide association study of chickpea reference set identifies genetic determinants of Pratylenchus thornei resistance
Source: Front Plant Sci. 2023 Mar 24;14:1139574. doi: 10.3389/fpls.2023.1139574 (PMC10080060; doi:10.3389/fpls.2023.1139574)
Supplement: Supplementary file 1 [file DataSheet_1.pdf]

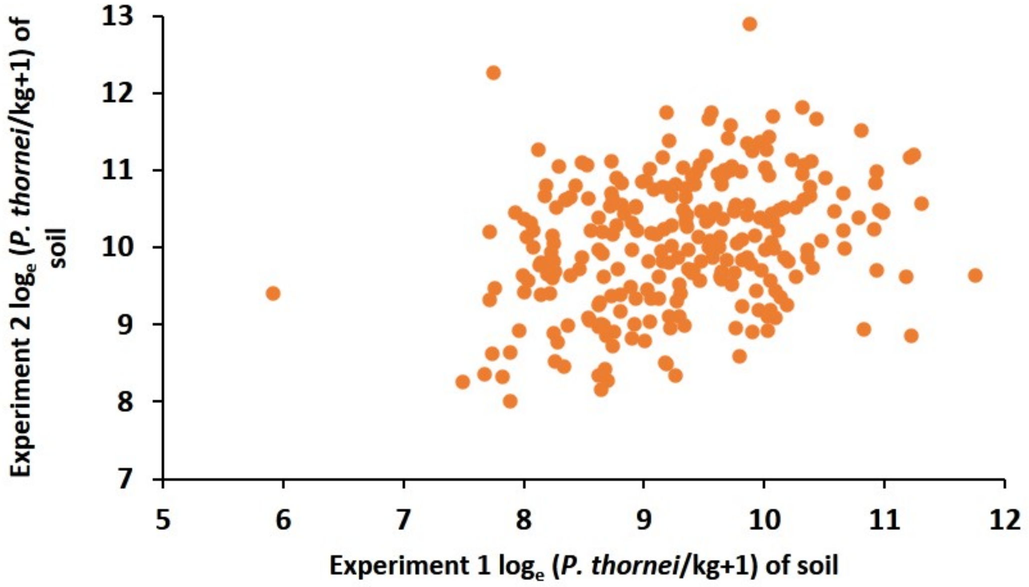

Supplementary Figure 1. Accessions means of *Pratylenchus thornei* population densities ($\log_e$ (*P. thornei*/kg +1) of soil) of Experiment 1 and 2 revealed a moderate correlation ($r= 0.3$, $P < 0.0001$, $n=265$) between the two experiments.

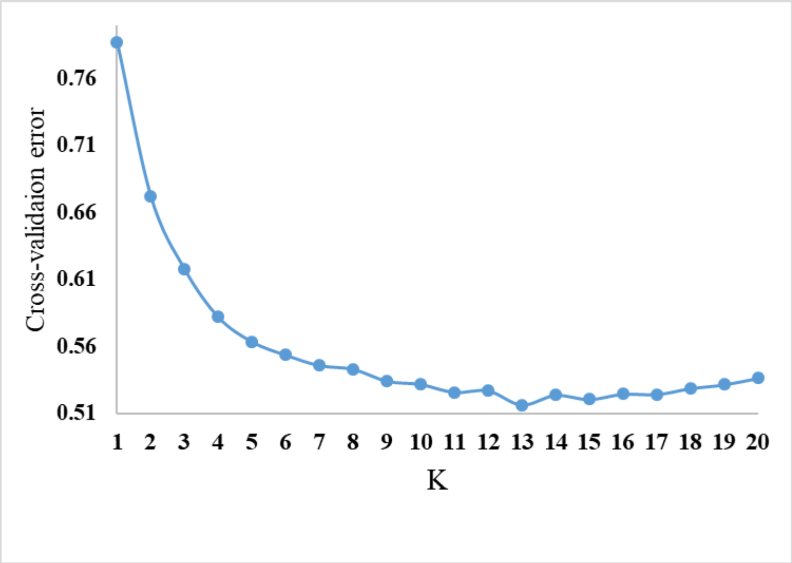

Supplementary Figure 2. Cross-validation (CV) plot for ADMIXTURE K=1 to 20, with CV = 10.

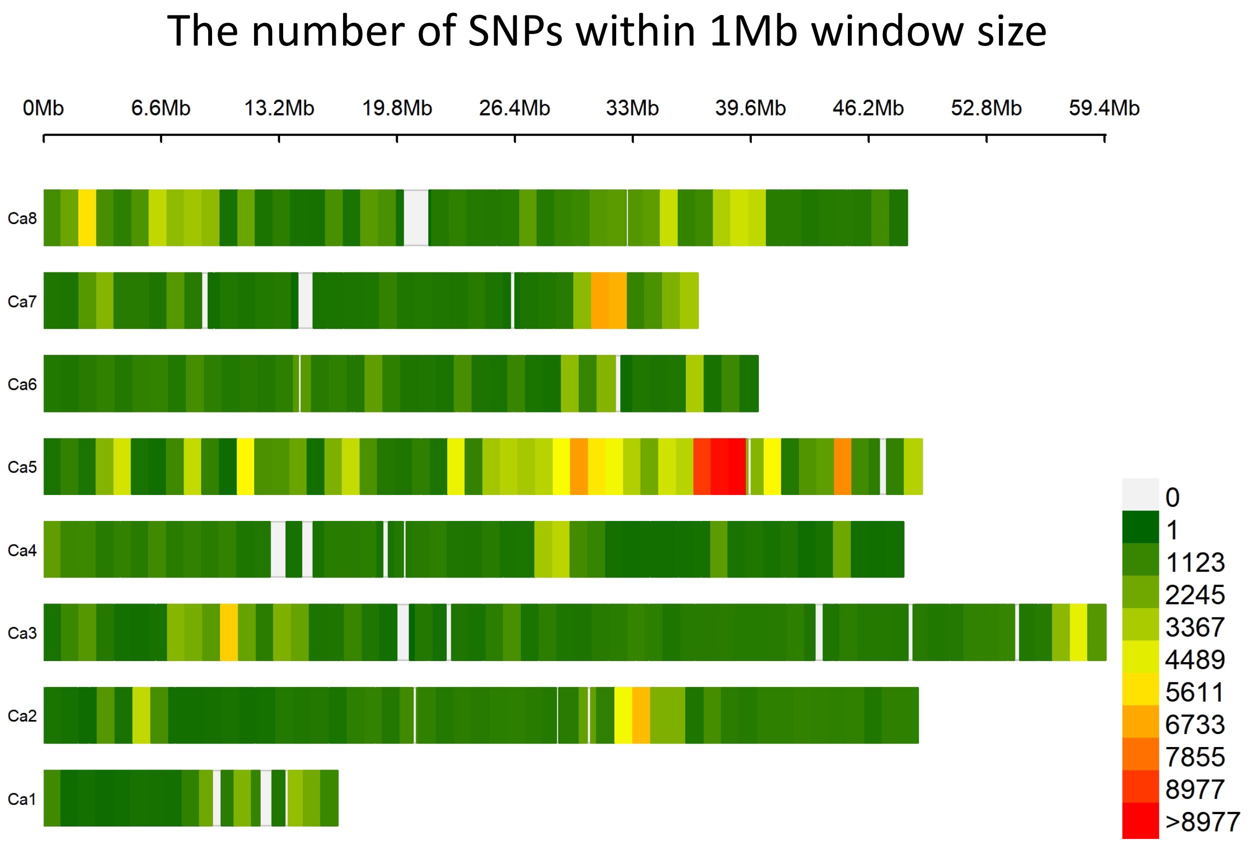

Supplementary Figure 3: SNP density plot across the 8 chromosomes of chickpea representing the number of SNPs within 1 Mb window size. The horizontal axis represents the chromosome length in Mb. Different colors correspond to SNP density.
